# SIRT6 mediates MRTF-A deacetylation in vascular endothelial cells to antagonize oxLDL-induced ICAM-1 transcription

**DOI:** 10.1038/s41420-022-00903-y

**Published:** 2022-03-04

**Authors:** Shan Huang, Tinghui Shao, Hong Liu, Qianyun Wang, Tianfa Li, Qianwen Zhao

**Affiliations:** 1 Key Laboratory of Targeted Intervention of Cardiovascular Disease and Collaborative Innovation Center for Cardiovascular Translational Medicine, Department of Pathophysiology, Nanjing Medical University, Nanjing, China; 2 Hainan Provincial Key Laboratory for Tropical Cardiovascular Diseases Research, Key Laboratory of Emergency and Trauma of Ministry of Education, Research Unit of Island Emergency Medicine of Chinese Academy of Medical Sciences, Department of Cardiology, the First Affiliated Hospital of Hainan Medical University, Haikou, China; 3 Department of Thoracic Surgery, the Third Affiliated Hospital to Soochow University, Changzhou, China

**Keywords:** Acetylation, Transcription

## Abstract

Oxidized low-density lipoprotein (oxLDL), a known risk factor for atherosclerosis, activates the transcription of adhesion molecules (ICAM-1) in endothelial cells. We previously showed that myocardin-related transcription factor A (MRTF-A) mediates oxLDL-induced ICAM-1 transcription. Here we confirm that ICAM-1 transactivation paralleled dynamic alterations in MRTF-A acetylation. Since treatment with the antioxidant NAC dampened MRTF-A acetylation, MRTF-A acetylation appeared to be sensitive to cellular redox status. Of interest, silencing of SIRT6, a lysine deacetylase, restored MRTF-A acetylation despite the addition of NAC. SIRT6 directly interacted with MRTF-A to modulate MRTF-A acetylation. Deacetylation of MRTF-A by SIRT6 led to its nuclear expulsion thus dampening MRTF-A occupancy on the ICAM-1 promoter. Moreover, SIRT6 expression was downregulated with oxLDL stimulation likely owing to promoter hypermethylation in endothelial cells. DNA methyltransferase 1 (DNMT1) was recruited to the SIRT6 promoter and mediated SIRT6 repression. The ability of DNMT1 to repress SIRT6 promoter partly was dependent on ROS-sensitive serine 154 phosphorylation. In conclusion, our data unveil a novel DNMT1-SIRT6 axis that contributes to the regulation of MRTF-A acetylation and ICAM-1 transactivation in endothelial cells.

## Introduction

Atherosclerosis, defined as the appearance of fat-laden plaques in the major arteries, is a prototypic form of coronary heart disease, which claims over half a million lives each year in the US alone [1]. Multiple risk factors including smoking, obesity, diabetes, hypertension, and hyperlipidemia, can lead to atherosclerosis [2]. Decades of research have conclusively demonstrated that atherosclerosis is primarily a pathology of chronic inflammation [3]. Indeed, a plethora of immune cell lineages can be detected within the atherosclerotic plaque and subsequently contribute to atherogenesis in animal models and in humans [4]. In order for the circulating leukocytes to trespass through the vasculature and into the plaque, a firm interaction has to be established between these cells and the endothelial layer [5]. Previous studies showed that the endothelium–leukocyte interaction is mediated by a group of intercellular adhesion molecules (ICAMs). ICAM-1, for instance, promotes the adhesion of monocytes, macrophages, and neutrophils to the endothelium and is upregulated in the human atherosclerotic plaques [6]. On the contrary, ICAM-1 deficiency prevents the progression of atherosclerosis, macrophage accumulation in the plaque, and vascular inflammation in mice [7, 8]. In endothelial cells, ICAM-1 can be transcriptionally activated by oxLDL which is a known risk factor for atherosclerosis [9]. The sequence-specific transcription factor NF-κB is believed to be a major activator of ICAM-1 transcription through directly binding to the proximal ICAM-1 promoter [10].

Myocardin-related transcription factor A (MRTF-A) belongs to the family of proteins initially identified as co-factors for the sequence-specific transcription factor SRF [11–13]. Unlike the founding member of this family Myocardin, which is exclusively expressed in muscle cells, MRTF-A is ubiquitously expressed in all tissues and cells [11]. Absence of MRTF-A is tolerated in embryogenesis and adulthood as evidenced by the fact that mice with germline MRTF-A deletion are born with Mendelian ratios and exhibit no overt phenotype under physiological conditions [14, 15]. Several lines of evidence point to a potential role for MRTF-A in atherogenesis. We have previously confirmed that MRTF-A is expressed in endothelial cells and interacts with NF-κB to mediate oxLDL-induced ICAM-1 transcription and leukocyte adhesion [16]. Minami et al. have demonstrated that MRTF-A deficiency in an *Apoe*^−/−^ background retards the process of atherosclerosis in mice [17]. Besides, population studies have identified a position correlation between MRTF-A polymorphisms and increased risk of coronary heart disease [18, 19].

Transcriptional activity of MRTF-A is regulated by several factors including its post-translational modifications. Previous studies have shown that MRTF-A can be subjected to phosphorylation [20], SUMOylation [21], ubiquitination [22], and acetylation [23]. Modifications of MRTF-A modulate its activity by impacting its stability, subcellular localization, and interactions with co-factors. In this study, we provide evidence to show the acetylation status of MRTF-A correlates with ICAM-1 expression in endothelial cells. Redox-sensitive repression of the lysine deacetylase SIRT6, mediated by DNA methylation, leads to augmentation of MRTF-A acetylation and ICAM-1 activation.

## Results

### SIRT6 mediates redox-sensitive deacetylation of MRTF-A

**Fig. 1:**
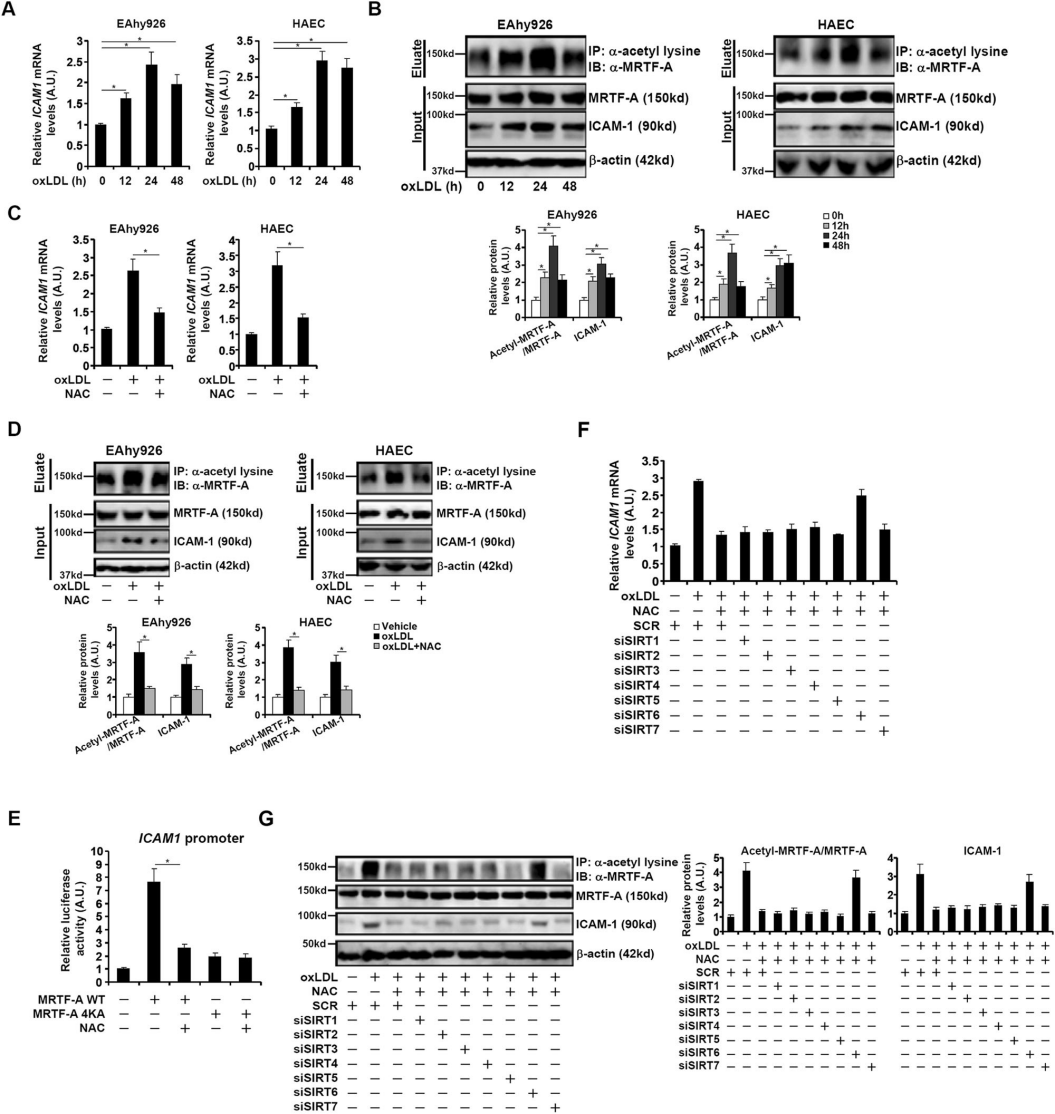
SIRT6 mediates redox-sensitive deacetylation of MRTF-A. **A, B** EAhy926 cells and primary human aortic endothelial cells (HAECs) were treated with or without oxLDL and collected at indicated time points. ICAM-1 expression was examined by qPCR (**A**). Immunoprecipitation was performed with anti-acetyl lysine (**B**). **C, D** EAhy926 cells and primary human aortic endothelial cells (HAECs) were treated with or without oxLDL and collected at indicated time points. CAM-1 expression was examined by qPCR **C**. Immunoprecipitation was performed with anti-acetyl lysine (**D**). **E** An ICAM-1 promoter-luciferase construct was transfected into EAhy926 cells with wild type or mutant MRTF-A expression construct followed by treatment with NAC. Luciferase activities were normalized by protein concentration and GFP fluorescence. **F, G** EAhy926 cells were transfected with indicated siRNAs followed by treatment with oxLDL and/or NAC for 24 h. ICAM-1 expression was examined by qPCR (**F**). Immunoprecipitation was performed with anti-acetyl lysine **G**. Error bars represent SD (*p < 0.05, one-way ANOVA). All experiments were repeated three times and one representative experiment is shown.

When cultured endothelial cells (EAhy926) and primary human aortic endothelial cells (HAECs) were exposed to oxLDL, ICAM-1 expression was upregulated (Figs. 1A, B). Paralleling ICAM-1 upregulation, there was a simultaneous upregulation of MRTF-A acetylation level whereas overall MRTF-A expression level was marginally affected (Fig. 1B). Interestingly, treatment with an antioxidant N-acetylcysteine (NAC) attenuated ICAM-1 induction by oxLDL and dampened MRTF-A acetylation (Figs. 1C, D). Consistently, over-expression of MRTF-A in endothelial cells potently augmented the ICAM-1 promoter activity, however, NAC treatment diminished activation of the ICAM-1 promoter. Mutation of a series of 4 lysine residues within MRTF-A that disables its acetylation [23] strongly crippled the ability of MRTF-A to trans-activate the ICAM-1 promoter and rendered MRTF-A unresponsive to NAC treatment (Fig. 1E).

Sirtuin family of lysine deacetylase plays a key role in regulating endothelial function. When individual sirtuin was depleted with siRNA (Fig. S1 for validation of knockdown efficiency and specificity), it was discovered that reducing the expression of SIRT6 reversed the effect of NAC treatment to restore oxLDL-induced ICAM-1 expression (Fig. 1F) and MRTF-A acetylation (Fig. 1G). Similar results were obtained with a second pair of SIRT6 siRNAs (Fig. S2).

### SIRT6 interacts with MRTF-A and deacetylates MRTF-A

**Fig. 2:**
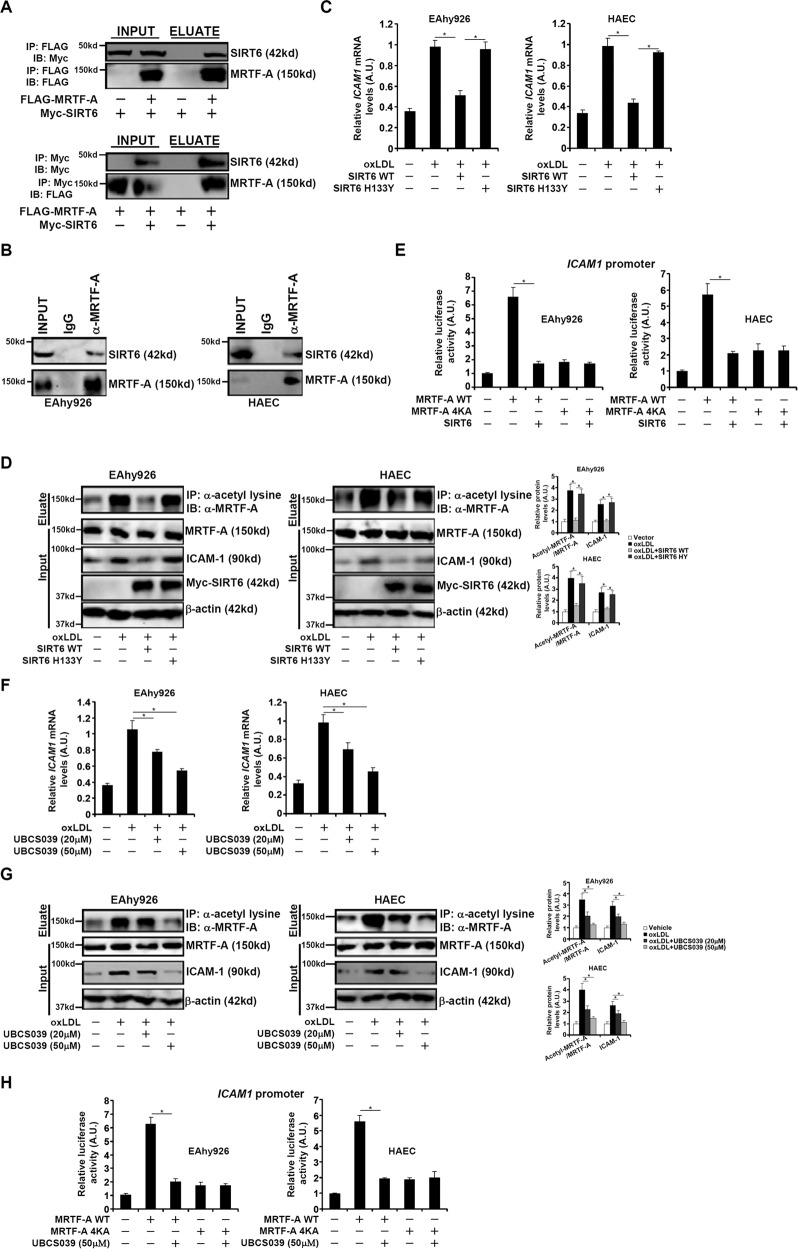
SIRT6 interacts with MRTF-A and deacetylates MRTF-A. **A** HEK293 cells were transfected with FLAG-tagged MRTF-A and Myc-tagged SIRT6. Immunoprecipitation was performed with anti-FLAG. **B** Immunoprecipitation was performed with EAhy926 cell lysates and HAEC cell lysates. **C, D** EAhy926 cells and primary human aortic endothelial cells (HAECs) were infected with lentivirus carrying wild type or mutant SIRT6 expressing vector followed by treatment with oxLDL. ICAM-1 expression was examined by qPCR (**C**). Immunoprecipitation was performed with anti-acetyl lysine (**D**). **E** An ICAM-1 promoter-luciferase construct was transfected into EAhy926 cells and HAECs with wild type or mutant MRTF-A expression construct and SIRT6 expression construct. Luciferase activities were normalized by protein concentration and GFP fluorescence. **F, G** EAhy926 cells and primary human aortic endothelial cells (HAECs) were treated with oxLDL in the presence or absence of UBCS039. ICAM-1 expression was examined by qPCR (**F**). Immunoprecipitation was performed with anti-acetyl lysine (**G**). **H** An ICAM-1 promoter-luciferase construct was transfected into EAhy926 cells and HAECs with wild type or mutant MRTF-A expression construct followed by treatment with UBCS039. Luciferase activities were normalized by protein concentration and GFP fluorescence. Error bars represent SD (*p < 0.05, one-way ANOVA). All experiments were repeated three times and one representative experiment is shown.

Next, we examined whether SIRT6 could interact with and deacetylate MRTF-A. Myc-tagged SIRT6 was co-transfected into HEK293 cells without FLAG-tagged MRTF-A; an anti-FLAG antibody precipitated Myc-SIRT6 only when FLAG-MRTF-A was present (Fig. 2A). Furthermore, Co-IP experiments also demonstrated that endogenous MRTF-A interacted with SIRT6 in endothelial cells (Fig. 2B). Transduction with lentivirus carrying SIRT6 WT, but not an enzymatically deficient SIRT6 (H133Y), dampened induction of ICAM-1 expression under oxLDL stimulation while at the same time attenuated MRTF-A acetylation in endothelial cells (Figs. 2C, D). Of note, SIRT6 appeared to be able inhibited ICAM-1 transactivation only in the presence of the wild-type MRTF-A but not the acetylation mutant MRTF-A (Fig. 2E). Similarly, treatment with a specific SIRT6 activator UBCS039 achieved equivalent effects as SIRT6 over-expression to diminish ICAM-1 expression and MRTF-A acetylation in a dose-dependent manner (Figs. 2F, G). Again, it was observed that the effect of UBCS039 was only evident with wild-type MRTF-A but not with the mutant MRTF-A (Fig. 2H).

### SIRT6-mediated deacetylation promotes nuclear expulsion of MRTF-A

**Fig. 3:**
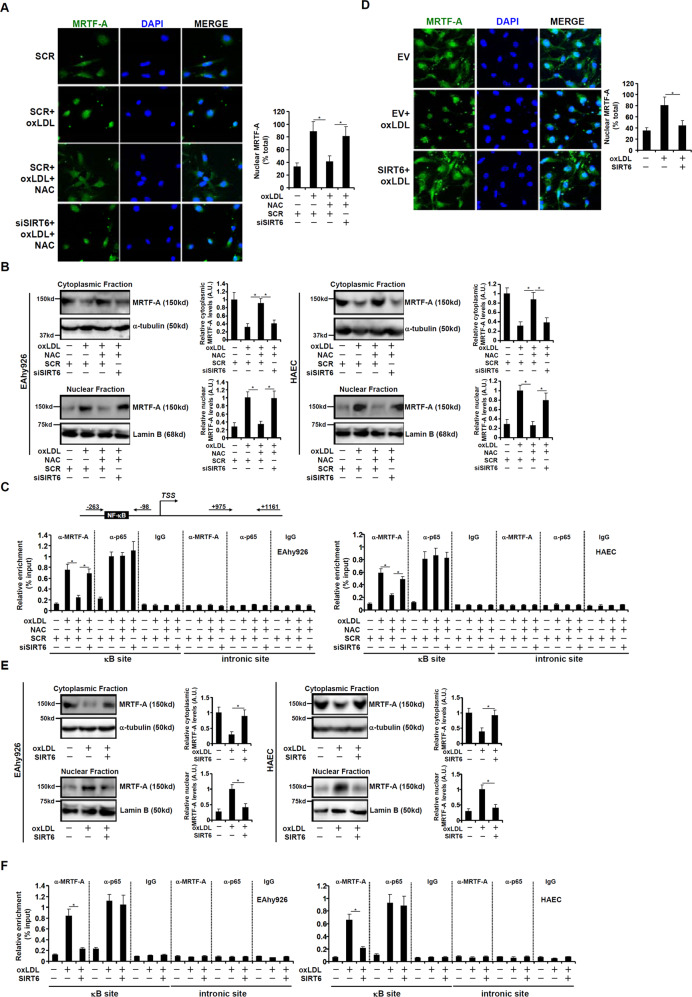
SIRT6-mediated deacetylation promotes nuclear expulsion of MRTF-A. **A** EAhy926 cells were transfected with a SIRT6 expression vector followed by treatment with oxLDL. Immunofluorescence staining was performed with anti-MRTF-A. **B** EAhy926 cells and HAECs were transfected with a SIRT6 expression vector followed by treatment with oxLDL. Cytoplasmic and nuclear proteins were extracted as described in Methods and MRTF-A protein in different fractions was detected by Western. **C** EAhy926 cells and HAECs were transfected with a SIRT6 expression vector followed by treatment with oxLDL. ChIP was performed with anti-MRTF-A, anti-NF-κB/p65, or IgG. **D** EAhy926 cells were transfected with indicated siRNAs followed by treatment with oxLDL and/or NAC for 24 h. Immunofluorescence staining was performed with anti-MRTF-A. **E** EAhy926 cells and HAECs were transfected with indicated siRNAs followed by treatment with oxLDL and/or NAC for 24 h. Cytoplasmic and nuclear proteins were extracted as described in Methods and MRTF-A protein in different fractions was detected by Western. **F** EAhy926 cells and HAECs were transfected with indicated siRNAs followed by treatment with oxLDL and/or NAC for 24 h. ChIP was performed with anti-MRTF-A, anti-NF-κB/p65, or IgG. Error bars represent SD (*p < 0.05, one-way ANOVA). All experiments were repeated three times and one representative experiment is shown.

We then explored the impact of SIRT6-mediated MRTF-A deacetylation in endothelial cells. Immunofluorescence staining showed that MRTF-A primarily resided in the cytoplasm of endothelial cells under normal conditions and oxLDL treatment prompted nuclear translocation of MRTF-A, which was largely prevented by NAC; SIRT6 knockdown, however, nullified the effect of NAC treatment and allowed oxLDL to stimulate MRTF-A nuclear accumulation (Fig. 3A). Cellular fractionation followed by Western blotting confirmed that reducing SIRT6 expression negated the blockade of MRTF-A nuclear translocation by NAC (Fig. 3B). We also used the occupancy of MRTF-A on the ICAM-1 promoter, assessed by ChIP assay, as a proxy to determine its nuclear accumulation. As shown in Fig. 3C, oxLDL enhanced occupancy of MRTF-A on the ICAM-1 promoter whereas NAC treatment abrogated MRTF-A recruitment; SIRT6 knockdown re-installed MRTF-A on the ICAM-1 promoter. By comparison, whereas oxLDL treatment augmented the occupancy of NF-κB/p65, the sequence-specific transcription factor essential for MRTF-A recruitment to the ICAM-1 promoter [16], neither NAC treatment nor SIRT6 knockdown affected p65 binding (Fig. 3C), indicating that MRTF-A recruitment might be the rate-limiting step for oxLDL-induced ICAM-1 transactivation.

In another set of experiments, we found that increasing SIRT6 expression, through lentiviral transduction, antagonized MRTF-A nuclear accumulation prompted by oxLDL treatment, as measured by immunofluorescence staining (Fig. 3D) and cell fractionation (Fig. 3E). Besides, SIRT6 over-expression also blocked the recruitment of MRTF-A to the ICAM-1 promoter without altering NF-κB/p65 binding (Fig. 3F). Taken together, these data suggest that SIRT6-mediated MRTF-A deacetylation may control its subcellular localization.

### SIRT6 is transcriptionally repressed by DNMT1 in endothelial cells

**Fig. 4:**
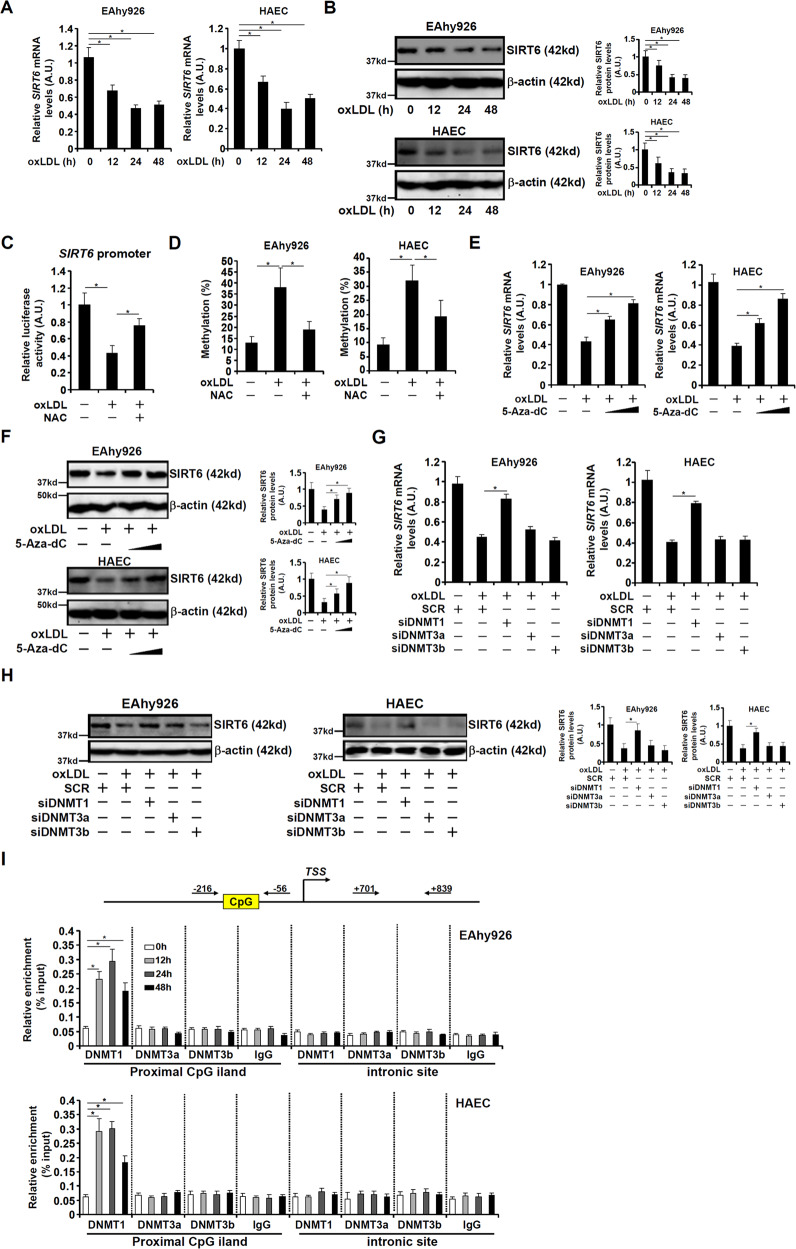
SIRT6 is transcriptionally repressed by DNMT1 in endothelial cells. **A, B** EAhy926 cells and HAECs were treated with or without oxLDL and collected at indicated time points. SIRT6 expression was examined by qPCR (**A**) and Western (**B**). **C** A human SIRT6 promoter construct was transfected into EAhy926 cells followed by treatment with oxLDL and/or NAC. Luciferase activities were normalized by protein concentration and GFP fluorescence. **D** EAhy926 cells and HAECs were treated with or without oxLDL for 24 h. DNA methylation was evaluated as described in Methods. **E, F** EAhy926 cells and HAECs were treated with oxLDL in the presence or absence of 5-Aza-dC for 24 h. SIRT6 expression was examined by qPCR (**E**) and Western (**F**). **G, H** EAhy926 cells and HAECs were transfected with indicated siRNAs followed by treatment with oxLDL for 24 h. SIRT6 expression was examined by qPCR (**G**) and Western (**H**). **I** EAhy926 cells and HAECs were treated with oxLDL and harvested at indicated time points. ChIP assay was performed with anti-DNMT1, anti-DNMT3a, anti-DNMT3b, or IgG. Error bars represent SD (*p < 0.05, one-way ANOVA). All experiments were repeated three times and one representative experiment is shown.

When endothelial cells were exposed to oxLDL, SIRT6 expression levels were downregulated (Figs. 4A, B). To determine whether SIRT6 expression was regulated at the transcriptional level, a SIRT6 promoter-luciferase construct was transfected into endothelial cells: oxLDL treatment repressed the SIRT6 promoter activity, which was normalized by NAC treatment (Fig. 4C). Numerous studies have pointed to promoter hypermethylation as a mechanism for SIRT6 gene repression [24–26]. Indeed, bisulfite assay demonstrated that oxLDL treatment stimulated SIRT6 promoter hypermethylation, however, NAC treatment neutralized the effect of oxLDL on SIRT6 promoter methylation (Fig. 4D). Further, treatment with a pan-DNA methyltransferase inhibitor 5-Aza-dC dose-dependently relieved the repression of SIRT6 expression by oxLDL (Figs. 4E, F). DNA methylation in mammalian cells is catalyzed by one of the three major DNA methyltransferases, DNMT1, DNMT3a, and DNMT3b [27]. Knockdown of DNMT1, but not DNMT3a or DNMT3b, canceled the repression of SIRT6 expression by oxLDL treatment in endothelial cells (Figs. 4G, H). Consistent with these observations, the ChIP assay showed that oxLDL treatment stimulated the recruitment of DNMT1 instead of DNMT3a or DNMT3b to the SIRT6 promoter region (Fig. 4I).

### Redox-sensitive phosphorylation of DNMT1 mediates SIRT6 repression in endothelial cells

**Fig. 5:**
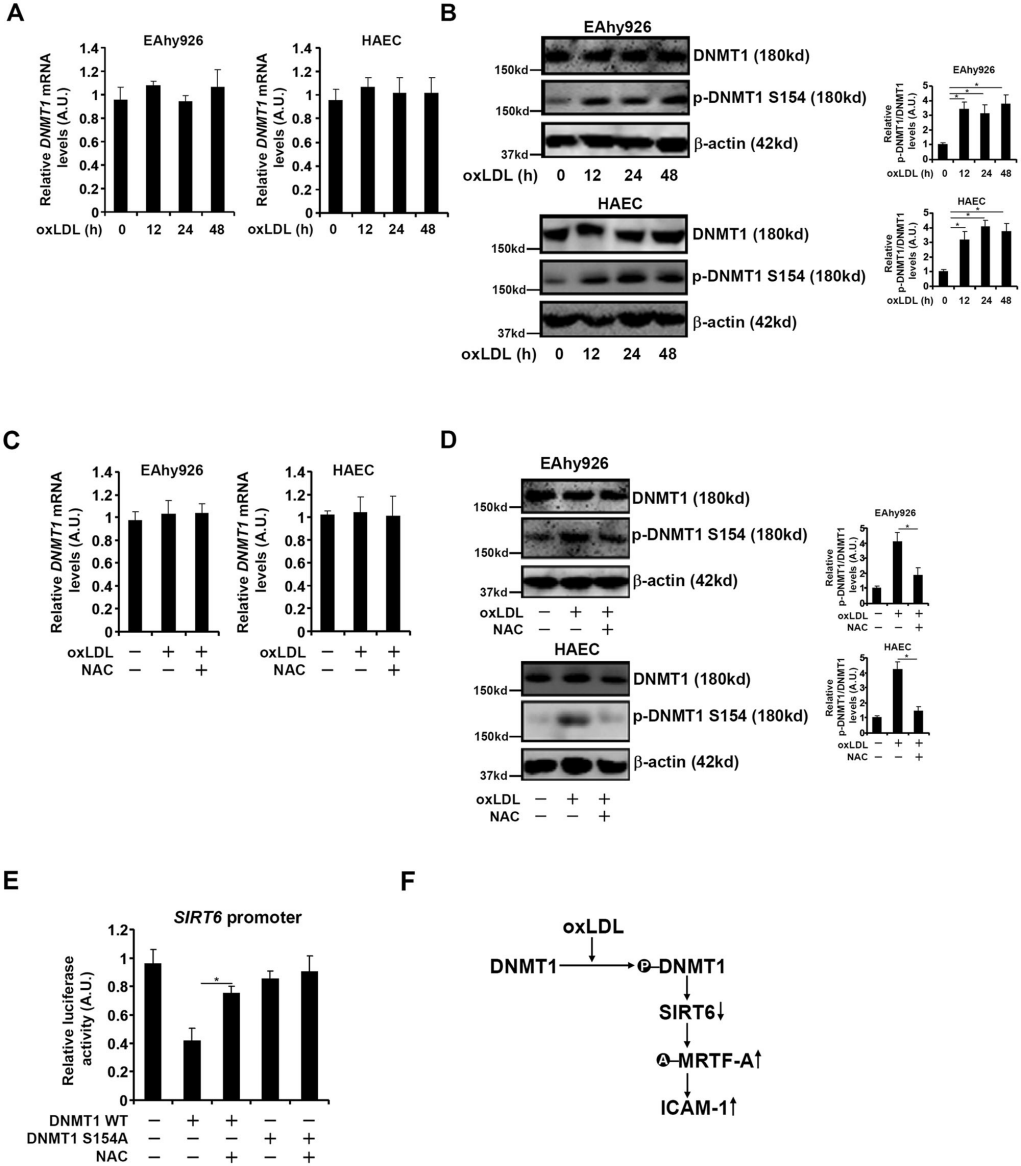
Redox-sensitive phosphorylation of DNMT1 mediates SIRT6 repression in endothelial cells. **A, B** EAhy926 cells were treated with or without oxLDL and collected at indicated time points. DNMT1 expression was examined by qPCR (**A**) and Western (**B**). **C, D** EAhy926 cells were treated with oxLDL and/NAC for 24 h. DNMT1 expression was examined by qPCR (**C**) and Western (**D**). **E** A human SIRT6 promoter-luciferase construct was transfected with EAhy926 cells with wild-type or mutant DNMT1 followed by treatment with NAC. Luciferase activities were normalized by protein concentration and GFP fluorescence. Error bars represent SD (*p < 0.05, one-way ANOVA). All experiments were repeated three times and one representative experiment is shown. **F** A schematic model.

According to the above research results, we determined the mechanism whereby DNMT1 contributes to SIRT6 repression by oxLDL in endothelial cells. Quantitative PCR (Fig. 5A) and Western blotting (Fig. 5B) showed that DNMT1 expression levels were not altered by oxLDL. However, DNMT1 phosphorylation (serine 154) was significantly upregulated by oxLDL treatment (Fig. 5B). In addition, NAC treatment, whereas failing to influence DNMT1 expression, lessened DNMT1 phosphorylation (Figs. 5C, D). To examine the functional relevance of oxLDL-induced DNMT1 phosphorylation, wild type DNMT1 and phosphorylation-deficient mutant (S154A) DNMT1 were transfected along with the SIRT6 promoter-luciferase construct into endothelial cells: WT DNMT1 repressed the SIRT6 promoter whereas NAC blocked the repression by WT DNMT1; S154A DNMT1 did not repress the SIRT6 promoter and was irresponsive to NAC treatment (Fig. 5E). Collectively, these data identify that redox-sensitive phosphorylation of DNMT1 may be involved in SIRT6 repression in endothelial cells.

## Discussion

Endothelial dysfunction is considered the linchpin of atherogenesis [28–31]. Endothelial cells undergo profound transcriptomic changes during atherosclerosis. Recently, single-cell RNA-seq experiments have demonstrated that upregulation of inflammation-related genes, including ICAM-1, in endothelial cells is a signature event in atherogenesis [32]. We have previously confirmed that MRTF-A is essential for oxLDL-induced ICAM-1 transcription in endothelial cells. In this study, we describe a DNMT1-SIRT6 axis that antagonizes oxLDL-induced ICAM-1 transcription by regulating MRTF-A acetylation and consequently nuclear accumulation (Fig. 5F).

We present evidence to show that SIRT6 antagonizes ICAM-1 transactivation by modulating MRTF-A activity. This observation is consistent with preliminary reports that implicate SIRT6 in the pathogenesis of coronary heart disease (CHD). Xu et al. have shown that SIRT6 deficiency in *Apoe*^−/−^ mice exacerbate atherosclerosis when fed a Western diet [33]. Of interest, the same study also demonstrates that SIRT6 deletion further increases the expression of VCAM-1, an adhesion molecule that can be transcriptionally activated by MRTF-A with similar functions as ICAM-1, in the arteries although the potential involvement was not determined. Recent research by Camici and colleagues has shown that endothelial-specific SIRT6 aggravates whereas endothelial-specific SIRT6 over-expression ameliorates the incidence and severity of stroke, one of the most common consequences of CHD [34]. Mechanistically, the authors propose that beneficial effects SIRT6 confers depend on the preservation of blood–brain barrier and survival of endothelial cells. It should be emphasized that although we and other researchers have arrived at the same conclusion that SIRT6 suppression may underscore the pathogenesis of atherosclerosis and, more broadly, coronary heart disease, the underlying mechanism may not entirely rely on MRTF-A. SIRT6 has been reported to inhibit the activity of NF-κB by either removing the active acetyl H3K9 marker from NF-κB target genes or by de-repressing the transcription of IκB [35]. In addition, SIRT6 can interact with and deacetylate FOXO1 to expel FOXO1 from the nucleus thus de-activating FOXO1 [36]. Because FOXO1 contributes to endothelial dysfunction and has been shown to directly bind to the ICAM-1 promoter and activate ICAM-1 transcription [37, 38], it is plausible to speculate that SIRT6 might modulate oxLDL-induced ICAM-1 expression by targeting FOXO1.

We have previously reported that MRTF-A deacetylation can be modulated by two lysine deacetylases under different circumstances. HDAC5 deacetylates MRTF-A in macrophages to ameliorate TNF-α induced pro-inflammatory mediators [39]. Alternatively, SIRT1 deacetylates MRTF-A in dermal fibroblasts to defy senescence induced loss of collagen type I expression [40]. Here we show that SIRT6 interacts with and deacetylates MRTF-A in vascular endothelial cells. Apparently, all three deacetylases target the same four lysine residues within the N-terminus of MRTF-A. This discrepancy could be accounted for several explanations. First, differential expression levels of HDACs have been noted in endothelial cells [41, 42]. Thus, the ability of a specific deacetylase to modulate MRTF-A is dependent on its availability. Second, the catalytic activities of HDACs are subjected to the regulation by intracellular redox status and metabolic intermediates [43–48]. It is possible that even if a specific deacetylase is expressed in endothelial cells in sufficient quantity it may be rendered inactive by the intracellular milieu. Third, HDACs themselves are targets of post-translational modifications. SIRT6, for instance, can be phosphorylated and nitrated with varying consequences [49–52]. Thus, whether MRTF-A can be targeted by a specific deacetylase is also determined by the cell type-specific post-translational modification machinery. These issues need to be carefully examined in future studies.

In summary, our data echo recent findings that targeting SIRT6 bears translational significance in the intervention of coronary heart disease. SIRT6 expression appears to be downregulated in the atherosclerotic plaque in humans and animal models [53, 54] and in cultured endothelial cells by oxLDL treatment, which can be reversed by the DNMT inhibitor 5-Aza-dc (Fig. 4). Because administration of 5-Aza-dc attenuates atherosclerosis in *Ldlr*^−/−^ mice [55], it is tempting to speculate that a combined regimen consisting of a DNMT inhibitor and a SIRT6 activator could be considered a novel therapeutic solution for patients with CHD.

## Methods

### Cell culture and transient transfection

Immortalized human endothelial cells (EAhy926) were maintained in DMEM supplemented with 10% FBS as previously described [56]. MRTF-A expression constructs [23, 57], SIRT6 expression constructs [58], DNMT1 expression constructs [59], ICAM-1 promoter-luciferase constructs [16], and SIRT6 promoter-luciferase construct [60] have been previously described. Oxidized LDL (oxLDL) was purchased from Sigma. UBCS039 and 5-Aza-dC were purchased from Selleck. Small interfering RNAs were purchased from Dharmacon: SIRT1 siRNA, TCGAACAATTCTTAAAGAT; SIRT2 siRNA, GAGGCCAUCUUUGAGAUCAGCUAUU; SIRT3 siRNA, ACUCCCAUUCUUCUUUCAC; SIRT4 siRNA, GGUACUGGGCUAGAAACUU; SIRT5 siRNA, GCCAAGUUCAAGUAUGGCA; SIRT6 siRNA, AAGAATGTGCCAAGTGTAAGA; SIRT7 siRNA, GCCUGAAGGUUCUAAAGAA; DNMT1 siRNA, GCCUCAUCGAGAAGAAUAU; DNMT3a siRNA, GCGUCACACAGAAGCAUAU; DNMT3b siRNA, UUGUUGUUGGCAACAUCUGAA. Forty-eight hours after transfection and reporter activity, cells were collected and measured by using a luciferase reporter assay system (Promega) as previously described [61, 62].

### Protein extraction and western blot

Whole-cell lysates were obtained by re-suspending cell pellets in the lysis buffer (50 mMTris pH 7.4, 150 mM NaCl, 1% Triton X-100) with freshly added protease inhibitor (Roche) as previously described [63, 64]. Nuclear proteins were prepared with the NE-PER Kit (Pierce) following the manufacturer’s recommendation. Cell lysates were incubated with specific antibodies at 4 °C overnight and then conjugated with Protein A/G-plus Agarose beads (Santa Cruz). Alternatively, FLAG-conjugated beads (M2, Sigma) were added to and incubated with lysates overnight. The precipitated immune complex was eluted with 3X FLAG peptide (Sigma) before being released by boiling with 1X SDS electrophoresis sample buffer. Proteins were separated by 8% polyacrylamide gel electrophoresis with pre-stained markers (Bio-Rad) for estimating molecular weight and efficiency of transfer to blots. Proteins were transferred to nitrocellulose membranes (Bio-Rad) in a Mini-Trans-Blot Cell (Bio-Rad). The membranes were blocked with 5% nonfat dry milk in Tris-buffered saline buffer (0.05% Tween 20, 150 mM NaCl, 100 mMTris-HCl pH7.4) at 4 °C overnight. Western blot analyses were performed with anti-ICAM-1 (Proteintech, 60299-1), anti-MRTF-A (Santa Cruz, sc-32909), anti-SIRT6 (Abcam, ab62739), anti-FLAG (Sigma, F3165), anti-MYC (Santa Cruz, sc-40), anti-DNMT1 (Santa Cruz, sc-20701), anti-phosphorylated S154 DNMT1 (Thermo Fisher, PA5-12963), and anti-β-actin (Sigma, A2228) antibodies. For densitometrical quantification, densities of target proteins were normalized to those of β-actin as previously described (Sun et al., 2020; Wu et al., 2020a). Data are expressed as relative protein levels compared to the control group which is arbitrarily set as 1.

### RNA isolation and real-time PCR

RNA was extracted by using the RNeasy RNA isolation kit (Qiagen). Reverse transcriptase reactions were performed using a SuperScript First-strand Synthesis System (Invitrogen) as previously described [65, 66]. Real-time PCR reactions were performed on an ABI Prism 7500 system with the following primers: *ICAM-1*, 5'-AGCGGCTGACGTGTGCAGTAAT-3' and 5'-TCTGAGACCTCTGGCTTCGTCA-3'; SIRT6, 5'-TGGCAGTCTTCCAGTGTGGTGT-3' and 5'-CGCTCTCAAAGGTGGTGTCGAA-3'; *DNMT1*, 5'-AGGTGGAGAGTTATGACGAGGC-3' and 5'-GGTAGAATGCCTGATGGTCTGC-3'. Ct values of target genes were normalized to the Ct values of a housekeeping control gene (18 s, 5'-CGCGGTTCTATTTTGTTGGT-3' and 5'-TCGTCTTCGAAACTCCGACT-3' for both human and mouse genes) using the ΔΔCt method and expressed as relative mRNA expression levels compared to the control group which is arbitrarily set as 1.

### Chromatin immunoprecipitation (ChIP)

Chromatin immunoprecipitation (ChIP) assays were performed essentially as described before [67, 68]. Briefly, chromatin in control and treated cells were cross-linked with 1% formaldehyde. Cells were incubated in lysis buffer (150 mM NaCl, 25 mMTris pH 7.5, 1% Triton X-100, 0.1% SDS, 0.5% deoxycholate) supplemented with protease inhibitor tablet and PMSF. DNA was fragmented into ~200 bp pieces using a Branson 250 sonicator. Aliquots of lysates containing 200 μg of protein were used for each immunoprecipitation reaction with anti-MRTF-A (Santa Cruz, sc-10768), anti-DNMT1 (Santa Cruz, sc-20701), anti-DNMT3a (Santa Cruz, sc-20703), or anti-DNMT3b (Santa Cruz, sc-20704). Precipitated genomic DNA was amplified by real-time PCR with the following primers: *ICAM-1* proximal promoter, 5'-CCCTGCCACCGCCGCC-3' and 5'-AGGGGCGGTGCTGCTTTCC-3'; *ICAM-1* intronic region, 5'-AATTCCAGAGCTGACTTATCC-3' and 5'-ATCTCAGGCTTTGTTGAGC-3'; *SIRT6* promoter, 5'-AACTCTGCGTGGCATTCAAA-3' and 5'-AAATGCGGGACACAGGCTAT-3'. A total of 10% of the starting material is also included as the input. Data are then normalized to the input and expressed as % recovery relative to the input as previously described [56, 69]. All experiments were performed in triplicate wells and repeated three times.

### Immunofluorescence microscopy

Endothelial cells were fixed with 4% formaldehyde. After being permeabilized with TBST (.25% Triton X-100, 150 mM NaCl, 50 mM Tris pH7.4), endothelial cells were blocked with 5% BSA, and incubated with indicated primary antibodies at 4 °C overnight. Several washes with PBS, cells were incubated with FITC-labeled secondary antibodies (Jackson) for 30 min. DAPI (Sigma) was added and incubated with cells for 5 min prior to observation. Immunofluorescence was visualized on a confocal microscope (LSM 710, Zeiss).

### SIRT6 promoter DNA methylation analysis

SIRT6 promoter methylation status was analyzed with bisulfite conversion followed by sequencing essentially as described previously [24, 25]. Briefly, genomic DNA isolated from EAhy926 cells was subjected to bisulfite treatment using the EZ DNA Methylation Gold Kit (ZymoResearch). The modified DNA was amplified and the PCR products were cloned into pCRII-TA vectors (Invitrogen) and sequenced using the Sanger sequencing.

### Statistical analysis

Sample sizes reflected the minimal number needed for statistical significance based on power analysis and prior experience. Two-tailed Student’s *t*-test (for comparison between two groups) or ANOVA with post-hoc Scheffe test (for comparison among three or more groups) was performed using an SPSS package. Unless otherwise specified, *p*-values < 0.05 were considered statistically significant.

## Supplementary information

online data

## Data Availability

All data generated or analyzed during this study are included in this published article are available from the corresponding author on reasonable request.
