# Supplementary material for: SIRT6 mediates MRTF-A deacetylation in vascular endothelial cells to antagonize oxLDL-induced ICAM-1 transcription
Source: Cell Death Discov. 2022 Mar 4;8:96. doi: 10.1038/s41420-022-00903-y (PMC8897425; doi:10.1038/s41420-022-00903-y)
Supplement: Supplementary file 1 — online data [file 41420_2022_903_MOESM1_ESM.docx]

**Huang S et al: SIRT6 mediates MRTF-A deacetylation in vascular endothelial cells to antagonize oxLDL induced ICAM-1 transcription**

**Online supplementary material**

**Fig.S1:** EAhy926 cells were transfected with indicated siRNAs. Gene expression levels were examined by qPCR.

**Fig.S2:** EAhy926 cells were transfected with indicated siRNAs followed by treatment with oxLDL and/or NAC for 24h. (A) ICAM-1 expression was examined by qPCR. (B) Immunoprecipitation was performed with anti-acetyl lysine.
